# *Profex*: a graphical user interface for the Rietveld refinement program *BGMN*

**DOI:** 10.1107/S1600576715014685

**Published:** 2015-08-29

**Authors:** Nicola Doebelin, Reinhard Kleeberg

**Affiliations:** a RMS Foundation, Bischmattstrasse 12, Bettlach, 2544, Switzerland; b Institute of Geological Sciences, University of Bern, Baltzerstrasse 1–3, Bern, 3012, Switzerland; c Institut für Mineralogie, TU Bergakademie Freiberg, Brennhausgasse 14, Freiberg, 09596, Germany

**Keywords:** Rietveld refinement, graphical user interface, *BGMN*, *Profex*

## Abstract

*Profex* is a graphical user interface for the Rietveld refinement program *BGMN*. Its interface focuses on preserving *BGMN*’s powerful and flexible scripting features by giving direct access to *BGMN* input files. Very efficient workflows for single or batch refinements are achieved by managing refinement control files and structure files, by providing dialogues and shortcuts for many operations, by performing operations in the background, and by providing import filters for CIF and XML crystal structure files. Refinement results can be easily exported for further processing. State-of-the-art graphical export of diffraction patterns to pixel and vector graphics formats allows the creation of publication-quality graphs with minimum effort. *Profex* reads and converts a variety of proprietary raw data formats and is thus largely instrument independent. *Profex* and *BGMN* are available under an open-source license for Windows, Linux and OS X operating systems.

## Abstract 2

*Profex* is a platform-independent open-source graphical user interface for the Rietveld refinement program *BGMN*.

## 1. Introduction   

Academic software often arises from the need of a researcher or engineer to solve a specific computational problem. The developer initially focuses on the implementation of the numerical algorithm in order for the software to provide the minimum required functionality. As at that time the user base is often limited to the developer and possibly a small group in the developer’s vicinity, convenience features such as a graphical user interface (GUI) and documentation are addressed with low priority. In contrast to commercial software, whose developers need to target the largest possible user base and thus provide an accessible and attractive package with the very first public release, it is quite common for academic software to still lack convenience features after years of public availability. Particularly, the development of a modern GUI takes considerable resources and does not contribute to the scientific value. Authors not pursuing commercial interests and providing their academic software for free may further not see sufficient benefit in a growing user base to justify the efforts to develop a modern, feature-rich and complete GUI, and instead provide a basic GUI or none at all. However, if the software reaches a critical popularity, independent developers with or without relations to the core developer team may decide to provide an improved GUI to facilitate the use of the software. As the primary focus of these developers is on improving the users’ experience rather than on enhancing the core functionality of the software, their motivation to create a GUI complying with modern standards and expectations is likely to be high.

Rietveld refinement (RR) software that is used to evaluate powder X-ray diffraction (XRD) data files requires various input data besides the measured raw scan, such as crystal structure information, instrument configuration information or an instrument resolution function, and a specification of the refinement strategy. Various academic RR programs, including the well established *Fullprof.2k* (Rodriguez-Carvajal, 2001 ▸), *GSAS* (Larson & Von Dreele, 1994 ▸) and *BGMN* (Bergmann *et al.*, 1998 ▸), read this information from one or several text files created by the user and provided to the core program upon execution. GUIs for these RR kernels facilitate the creation of these input files, launch the refinement process, and present the diffraction pattern and refinement results to the user. Two fundamentally different approaches can be followed for handling input text files; the GUI may provide a low level of abstraction by allowing the user to directly modify the text files while also providing supporting features (*e.g.* text blocks, syntax highlighting, syntax checker, wizard dialogues) or a high level of abstraction by completely shielding the user from the text files and providing input elements (check boxes, dropdown menus, buttons, spin boxes) for all options. The former approach preserves the flexibility of the input text files, which is particularly important if the RR kernel supports scripting, but it also imposes a steeper learning curve on the user. The latter approach is generally perceived as more intuitive, but it limits scripting flexibility and bears a certain risk for inexperienced users who might over-process the data by activating too many and excessively complex refinement parameters.

A representative of the former approach is *Profex*, an alternative modern GUI for the RR program *BGMN* developed as an independent project by the corresponding author. This article provides an overview of the program’s features and some background information on design strategies, programming technique, and license and availability.

## 2. *BGMN*   

The RR software *BGMN* was developed by Jörg Bergmann as a non-institutional project starting in the early 1990s. The code was written in C under OS/2, later in a Linux environment, and executables for Windows (Microsoft, 2015 ▸), OS X (Apple, 2015 ▸) and Linux were compiled. The primary structure consists of executables for Monte Carlo modelling (*GEOMET*), de-convolution (*VERZERR*) and interpolation (*MAKEGEQ*) of the instrumental peak profiles, and of the RR kernel *BGMN*. In parallel, software for peak search (*EFLECH*) and indexing (*INDEX*) have been developed (Bergmann & Kleeberg, 1999 ▸). All input/output of the programs is organized *via* ASCII text files. Control and structure files apply a special interpreter language, enabling a very flexible description and formulation of structure models as well as the formulation of constraints and external calculation tasks.

The principle for all profile fitting procedures in the *BGMN* suite is a strict application of a convolution of wavelength-, geometry- and sample-related influences on the peak profiles (Bergmann *et al.*, 1998 ▸). The method of Monte Carlo simulation allows a reliable description of complex geometrical aberration profiles of laboratory diffractometers (Bergmann *et al.*, 2000 ▸). Only the sample-related peak broadening parameters are refinable; the instrumental ones must be predefined and are kept fixed in an RR procedure.

A very important characteristic of the *BGMN* software can be seen in its extremely stable convergence behaviour. Together with the carefully designed minimization algorithm, some special features in the software strongly stabilize the refinements. Especially, a built-in refinement strategy releases user-defined refined parameters sequentially in order to start with a robust set of parameters and activate complex anisotropic models like corrections of preferred orientation in later stages of the refinement. The refinement strategy also de-activates such models when not enough information (intensity, number of peaks, peak separation) can be found in the diffraction pattern. Thus, the *BGMN* software can be used without application of a user-defined or explicitly ‘learned’ refinement strategy, a big advantage for the routine task of quantitative phase analysis (QPA).

The main strength of *BGMN* can be seen in the powerful structure description language. This tool allows the formulation of models for the statistical description of stacking faults (Ufer *et al.*, 2008 ▸) and for one-dimensional diffraction patterns from oriented samples (Ufer *et al.*, 2012 ▸). Moreover, parametric refinement may be performed by the latest versions of *BGMN*. Such tasks demand free programmability of both the structure and task control files. Accordingly, the programming of such models is not straightforward and requires deep knowledge of the *BGMN* keywords, variables and predefined functions.

The general use of *BGMN* is realized by editing the input text files (structure descriptions and control files), starting the executables from the system command line, and evaluating the output files (diagrams, peak lists, refined parameters) by using editors or external software. In the original distributions the software comes with a manual and a number of basic example files and structure models, to be copied as file package.

### 2.1. *BGMNwin* interface   

The *BGMN* distribution includes a basic GUI named *BGMNwin* (Bergmann *et al.*, 2002 ▸), which is written in the Java programming language (Oracle, 2015 ▸) and follows a strict paradigm of low GUI abstraction level. It gives access to all *BGMN* input and output text files, including refinement control, structure and device files, and executes the core programs for refinement and calculation of peak profile functions. The handling of the input files is supported by a context-sensitive help function, opening a short description of the variables when marked in the text window. The measured and refined intensities are displayed graphically during the refinement and can be rendered to a pixel file for export. *BGMNwin* provides little more than the minimum functionality required to edit input files, launch the refinement and verify the results.

### 2.2. *AUTOQUAN*   

*AUTOQUAN* (Taut *et al.*, 1997 ▸) was developed as a GUI for the use of *BGMN* in routine QPA. The structure of the software follows the principle of shielding the user completely from any file handling and editing. Consequently the program can be used without training in using *BGMN* variables and file syntax. Crystal structure models are stored in a database system and the files necessary for running *BGMN* are created just temporarily and invisibly for the user. The structures primarily distributed with the database cannot be modified by the operator, but new entries can be created by copying and modification or by reading of standard *BGMN* structure files. Standard microstructure-related peak broadening models (isotropic and anisotropic crystallite size, micro-strain) can be chosen temporarily without changing the database entries. Tools for routine QPA like the application of internal standards or batch processing of sample series are provided. In standard database entries only those parameters may be refined that are essential for QPA, *i.e.* scale factors, lattice parameters, site occupation, peak profiles and preferred orientation models. Atomic coordinates and temperature factors are not accessible for refinement. Thus, the application is strictly limited to phase quantification; structure refinement is not supported. The import filter of raw data supports mainly the standard *BGMN* readable and some proprietary formats of Seifert Analytical X-ray. *AUTOQUAN* is a commercial software product.

## 3. *Profex*   

*Profex* was developed with the incentive of creating an efficient GUI for text-file-based academic RR programs that would not shield the user from editing text files but provide support through all stages of a Rietveld refinement from importing the raw scan to setting up the refinement control file and structure files, launching the refinement, and visualizing and exporting refinement results. *Profex* was initially developed as a frontend to *Fullprof.2k* (Rodriguez-Carvajal, 2001 ▸), which is loosely reflected by the application name, and was later enhanced to interact with *BGMN* (Bergmann *et al.*, 1998 ▸). Although *BGMN* has become the main target, legacy support for *Fullprof.2k* is still included and actively maintained, although with low priority. Key design features include an efficient workflow, preserving *BGMN*’s powerful scripting features, platform independence, and independence of diffractometer type and proprietary file format. The latter was achieved by implementing extensive support for a large number of raw data file formats from various instruments. The fundamental GUI approach is similar to *BGMN*’s native user interface *BGMNwin* in that the user edits input files in a text editor. However, *Profex*’s various additional and more refined features provide a significant improvement in terms of usability and efficiency.

The program *Profex* is entirely written in the C++ programming language using the Qt toolkit (http://www.qt.io/). It makes use of the *QuaZIP* (Tachenov, 2015 ▸) and *zlib* (Gailly & Adler, 2014 ▸) libraries to access compressed raw data file formats. Both *Profex* and *BGMN* are available on the major desktop operating systems Microsoft Windows (Microsoft, 2015 ▸), Apple OS X (Apple, 2015 ▸) and Linux. The source code of both programs is licensed under the GNU General Public License version 2 or later (Free Software Foundation, 1991 ▸) and is made available for download on the respective web sites at http://profex.doebelin.org and http://www.bgmn.de. Bundle archives containing *Profex*, *BGMN*, the user manuals for both programs in PDF format, a default set of crystal structure files and several examples of instrument configuration files are available for download on the *Profex* web site for Windows and OS X.

**Figure 1:**
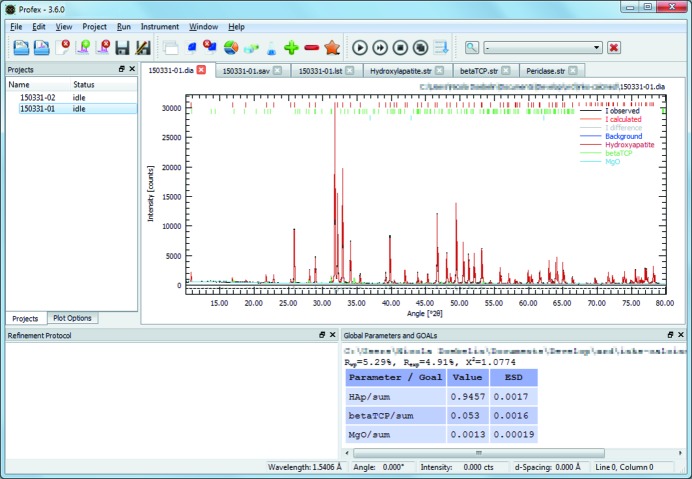
The *Profex* main window, showing the central plot display area with tabs of open text editors at the top, as well as several lists and output consoles docked and stacked left and below the plot display area.

The central element of *Profex*’s user interface is the plot display area, which is used to draw raw data scans and refined patterns (Fig. 1 ▸). The GUI follows a paradigm of low level of abstraction and therefore uses text editors to access and edit all refinement input files. These text editors open in tabs behind the plot display area and provide, in addition to the standard text editor functions, features specifically for *BGMN* input files. Various additional GUI elements are implemented as dockable widgets. These include the list of open projects, the list of scans in a project, the refinement protocol console, various results tables and the context help window. These dockable widgets are by default arranged around the central plot display, but they can be rearranged, closed, stacked on top of each other, or detached and placed as floating widgets on a second screen. The GUI can thus be customized in order to avoid clutter. Most of *Profex*’s functions are accessible from the menu bar, buttons in the tool bar, the mouse cursor’s context menu and keyboard shortcuts, which enables efficient workflows for advanced users while preserving intuitive use for novice users. Successful application of *Profex* by independent users was demonstrated for example by Pujari-Palmer *et al.* (2015 ▸) and Bobrovs *et al.* (2015 ▸).

### 3.1. Data file handling   

One of *Profex*’s strong features is its extensive support for raw data file formats. Considerable effort was made to support a large number of common powder XRD file formats by all major instrument manufacturers. Depending on the availability of file format documentation, the level of support ranges from basic reverse-engineered to complete support based on the manufacturer’s file format specification. An overview of supported formats with information on the level of support is given in Table 1 ▸. Level A and B supported formats read multi-range scans, whereas level C supported formats may not read multi-ranges correctly. A generic ASCII file format is also supported. It expects the first column to contain 2θ angle data and all following columns to contain intensity data for one range per column. Table 1 ▸ shows which characters are interpreted as field separators and comment signs.

Several raw data files can be inserted into the same plot display and stacked vertically and horizontally for easy comparison. The plot display area allows the user to interact with the imported scans in an intuitive manner. The scan can be zoomed and panned, the intensity axis can be scaled linearly, by log_10_ or by square root, and various mouse cursors are available to inspect positions of *K*α_1_, *K*α_2_ and *K*β peaks (optionally tungsten lines W *L*α_1_ and W *L*β_1_) and statistical counting noise.

Once imported and set up, scans can be exported from *Profex* to a variety of output file formats (Table 2 ▸). A batch conversion feature is available to convert any number of raw files to one of the output formats, except for pixel and vector images.

**Figure 2:**
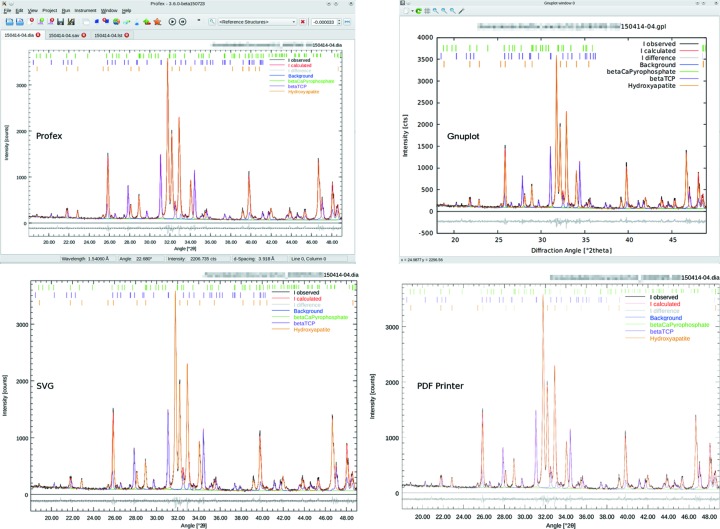
The on-screen graph display in *Profex* (top left) is accurately exported to *Gnuplot* (top right) or SVG format (botton left, rendered with *Inkscape*), or printed to PDF format (bottom right) for publication-quality graph export and further editing.

After a successful refinement the plot window displays observed, calculated, background, difference and phase contribution intensities, *hkl* tick marks, and a legend. The visibility of these elements can be adjusted individually to avoid cluttering of the graph display. Hovering the mouse cursor over an *hkl* tick mark will display the peak’s Miller indices, phase name and texture factor. While all output formats shown in Table 2 ▸ export intensity curves, several of them do not support the export of *hkl* tick marks, the legend, file names and axis labels. An accurate representation of the plot display on screen can be obtained with the pixel format (PNG), vector format (SVG) and *Gnuplot* script (Williams & Kelley, 2015 ▸). Scalable vector graphics (SVG) is the most versatile format for creating publication-quality figures. It preserves all information shown on screen (Fig. 2 ▸) and allows editing of all elements including font sizes, line widths, line and font colours and styles, positions of the legend and axis labels *etc*. in a vector drawing program such as *Inkscape* (Bah *et al.*, 2015 ▸), *Adobe Illustrator* (Adobe Systems, 2015 ▸) and *CorelDRAW* (Corel, 2015 ▸). These powerful graphics conversion features may even be useful for users who prefer to use different RR programs, provided their file format is supported for import by *Profex*.

### 3.2. Internal structure and device file databases   

*BGMN* reads crystal structure information from *BGMN*-specific structure files (STR file, extension *.str), which provide all structural information in a custom text format. A selection of STR files is provided for download on the *Profex* and *BGMN* web sites (http://profex.doebelin.org; http://www.bgmn.de). Similarly, *BGMN* retrieves peak profile information from a binary file with the extension *.geq that contains the peak shape computed from the instrument’s fundamental parameters. *Profex* uses directories containing a collection of structure and device files as internal databases. Files found in these folders can be added to a refinement project from a convenient dialogue. A default set of structure and device files is also included in the *Profex–BGMN* bundles offered for download. However, instead of accessing the directories stored inside the bundle, the structure and device databases can also be located on a network shared drive as a central repository accessed by multiple users. In a multi-user environment this setup allows for very easy maintenance; new structures or instrument configurations added by one user immediately become available to all users accessing the same repository.

### 3.3. Control, structure and results file management   

Manually creating and managing a refinement project for *BGMN* requires several steps of user interaction: (i) most users will need to convert the raw data file to a format supported by *BGMN*, as it does not import modern raw files such as Bruker RAW or BRML, or PANalytical XRDML files directly; (ii) creating a control file and copying all referenced structure and device files from a repository to the working directory; (iii) adjusting GOALs^1^ for calculation of phase quantities; and (iv) adjusting all input and output file names in the control file to the raw data file’s base name. This substantial amount of work is basically eliminated by *Profex*’s control file management features. The device configuration and all structure files can be selected from a dialogue, and *Profex* will perform steps (i) to (iv) mentioned above in the background. A control file will be created and the referenced file names will be adjusted, device and structure files will be copied into place, GOALs will be managed, and the raw data file will be converted if the native format is not supported by *BGMN*. User input is reduced to selecting the instrument configuration from a menu and selecting all phases to be refined from a list. *Profex* also indexes all structure files found in the local repository and allows the display of a phase’s *hkl* lines as a rudimentary means of phase identification prior to adding it to the refinement.

When a phase is added to a refinement project as described above, the corresponding structure file is automatically copied from the repository to the working directory. If necessary, line endings are converted to the target platform to avoid *BGMN* error messages. Once copied, the structure file can safely be edited for the refinement without altering the source file in the repository. *Profex* supports editing of structure files in several ways: (i) a button or short cut opens all structure files referenced in a control file in text editors, (ii) the text editors feature syntax highlighting, and (iii) the mouse cursor’s context menu allows toggling parameters from ‘fixed’ to ‘refine isotropically’ and, if applicable, to ‘refine anisotropically’.

After launching the refinement process from within *Profex*, *BGMN*’s console output will be displayed in the refinement protocol window and the plot will be updated after each refinement cycle. After convergence the results file will be opened in a text editor and a summary of global and local GOALs and parameters will be shown in tables. Global GOALs and parameters contain refined sample-specific parameters such as normalized phase quantities, sample height displacement and zero offset. Local GOALs and parameters contain phase-specific refinement results such as unit-cell dimensions, mean crystallite sizes, micro-strain, atomic coordinates and atomic site occupancies.

### 3.4. Batch processing   

The basic features of setting up and launching a refinement in *Profex* as described above allow for a very efficient workflow when processing a single data set. However, the refinement of a large number of similar data sets from replicate determinations would require substantial amounts of user input, as a separate control file would have to be created for each raw data file even though all files were measured with the same instrument configuration and the same phase content would be expected. Refinement of replicate data sets is simplified by *Profex*’s batch refinement features. Lists of raw data files can be loaded simultaneously into separate projects. The number of files to be loaded and processed in a batch refinement is only limited by the computer’s memory capacity. The authors successfully processed batches of more than 600 data sets. After creating a control file for the first project, the same control file can be applied to all open projects. Input and output file names will be adjusted automatically to the file name of the respective raw data file. Each project will access the same instrument configuration and structure files and thus start the refinement from identical conditions. Starting a batch refinement will refine all projects sequentially. It is also possible to start single refinements while other single or batch refinements are in progress. However, only one batch refinement can be started at a time. Single and batch refinements running in parallel can be stopped and restarted individually. The number of CPU cores to be used can be assigned to each refinement individually in order to manage the computer’s resources in a reasonable way.

Owing to the sophisticated multi-threaded handling of batch and parallel refinements, a single instance of *Profex* remains usable and responsive even during time-consuming refinements.

### 3.5. Exporting results   

The results of a converged refinement are written to a results file (file extension *.lst) which is automatically opened or updated in a text editor. A summary of global and local GOALs and parameters is furthermore shown tabulated in separate windows to allow for efficient verification of the results and quality of fit. These summarized global and local GOALs can be exported into separate spreadsheet files (CSV format) for further evaluation in a spreadsheet program. The export feature will merge global and local GOALs of all open projects into one spreadsheet file for global and one for local GOALs. This allows for easy evaluation of series of samples, for example calculations of mean values and standard deviations of batch refinements, without having to manually gather the results into one spreadsheet. The sorting feature of the spreadsheet program may be helpful to change the order of exported GOALs for evaluation.

Any calculated or refined parameter declared as a GOAL can be exported using the features described here. If the GOAL is declared in the refinement control file, it will automatically be exported with the global GOAL export function. Export of local GOALs, *i.e.* GOALs declared in structure files, can be configured in *Profex*’s preferences dialogue so as to only export results of interest.

### 3.6. Creating structure and device files   

If no crystal structure file (STR) for a specific phase is available in the structure database, it has to be created by the user. The format of STR files differs fundamentally from the crystallographic information file (CIF) format (Hall *et al.*, 1991 ▸), although both formats serve the same purpose of providing a description of a crystal structure. Manual translation of CIFs to STR files is a tedious and error-prone process, but it is vastly simplified by using *Profex*’s CIF import feature, which provides semi-automatic conversion of CIFs to STR files. In many cases CIFs do not contain all the information required for STR files and user input is required to assign the space group number, Hermann–Mauguin symbol or unit-cell setting. The resulting STR file may still be lacking information after the missing space group information has been provided (typically Wyckoff symbols or correct element symbols may still be missing), but owing to the dialogue-guided user interaction the efforts for conversion and risk for errors are reduced drastically.

Users of the commercial ICDD PDF-4+ (ICDD, 2014 ▸) database can export crystal structure data in a proprietary XML format, which can then be converted to STR files using *Profex*’s ICDD XML import feature. ICDD XML files are usually complete in that all information required for STR files is contained. If any user input is required at all, it is limited to selecting the correct Hermann–Mauguin symbol.

*BGMN* requires a precise description of the instrument configuration to calculate the shape of the peak profile. The description is provided in a text file which is then processed by *BGMN*-related programs to compute and interpolate the peak profile. *Profex* provides a text editor to view and edit the instrument configuration files and launches the Monte Carlo simulations and interpolations. The text editor features basic syntax checking and creates a template control file with monochromator and wavelength distribution information for the instrument. As with refinement control files, the low level of abstraction requires the user to edit text files, but it preserves the flexibility of *BGMN*’s syntax and scripting language. A number of configuration files for various types of instruments are provided with the *Profex* download archive. Many of these files contain elaborate comments and explanations and serve as a good starting point for a new instrument configuration.

### 3.7. Chemical composition   

**Figure 3:**
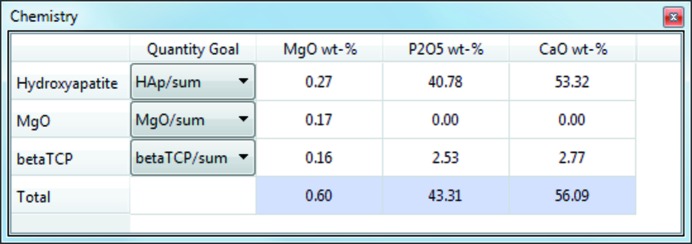
Chemical compositions are calculated from structural information and phase quantifications.

**Figure 4:**
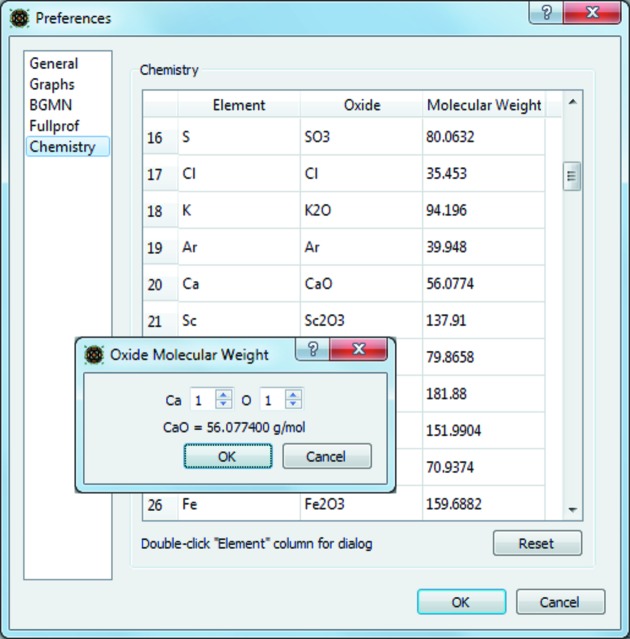
The oxide forms used to report chemical compositions can be customized.

The chemical composition of a refined crystalline phase can be calculated from the atomic site occupancies, site multiplicities and atomic weight of the site’s atomic species. *Profex* extracts this information from the project results file for all refined phases and computes the chemical composition using a hard-coded table of atomic weights. The compositions are then normalized by the refined quantities of the phases and presented in weight percent of the oxide in a table entitled ‘Chemistry’ (Fig. 3 ▸). The oxide forms can be customized to the user’s preference (Fig. 4 ▸). The as-calculated compositions should match with chemical analyses reporting oxide weight percentages (*e.g.* X-ray fluorescence), provided the sample was free of amorphous phases and atomic site species and occupancies were refined accurately.

Chemical compositions can be exported to a spreadsheet file for further evaluation in a spreadsheet program.

### 3.8. Refinement presets   

In situations when refinement results are to be compared with previously refined results, as for example in periodic quality control measurements or long-term experiments with periodic sample retrieval, it may be important to employ precisely the same refinement strategy each time so as to minimize strategy-related bias within the series. Creating a control file and applying a previously established refinement strategy the standard way bears the risk of incomplete adaptation and thus unnoticed differences among the strategies.

A robust approach for reoccurring standard refinements is given by *Profex*’s refinement preset feature. A manually created refinement can be saved as a preset and later be applied to a new raw data file with minimum effort. The preset stores the control file, instrument configuration file, a background file (if used) and all modified structure files in a repository. Applying the preset to a new raw data file creates a precise replicate of the refinement scenario established in the first place. Checksums are used to verify the validity of the preset files and to expose modifications after creation of the preset.

### 3.9. Context help   

Novice users may find it hard to memorize the meaning of all parameters in structure and control files, and also to understand the options for anisotropic refinements of certain parameters. The context help feature shows a description of the parameter the text cursor is placed on in the context help window. The text of the descriptions was adopted from *BGMNwin*’s context help.

## 4. Summary   

This article provides a general overview of *Profex*, which, in combination with *BGMN*, provides a comprehensive tool for Rietveld refinement of powder XRD data. *Profex*’s user interface focuses on a low level of abstraction to preserve *BGMN*’s powerful and flexible scripting features. Instrument independence is obtained by providing extensive support for proprietary raw data formats. Various supporting features result in an efficient workflow for refinements of single data sets or batch refinements of multiple data sets. Various export features allow for easy evaluation of refinement results and creation of publication-quality graphs. *Profex* is platform independent and freely available under a liberal open-source license.

## Figures and Tables

**Table 1:** Raw data file formats supported by *Profex*’s import feature Levels of support: A = full support based on format specification, B = good support reverse engineered, C = basic support reverse engineered.

Manufacturer	Extension	File format	Version	Level of support
Bruker	*.raw	Binary	V1, V2, V3, V4	A
Bruker	*.brml	XML	Compressed archive, single XML file with XML data container, single XML file with binary data container	B
PANalytical	*.xrdml	XML	1.01.5	A
Philips	*.rd	Binary		C
Philips	*.udf	ASCII		C
Seifert/FPM	*.val	ASCII		B
Rigaku	*.bin	Binary		C
Rigaku	*.dat, *.rig, *.dif	ASCII		C
Rigaku	*.raw	Binary		C
MDI Jade	*.xml	XML		C
MDI Jade	*.dif	ASCII		C
Stoe	*.pro	ASCII		C
Stoe	*.raw	Binary		C
Generic	*.xy	ASCII	Field separators: ; : , space tab	A
			Comment signs: ! % / #	
*BGMN*	*.dia	ASCII		A
*Fullprof.2k*	*.prf	ASCII	Only PRF = 3	A
*Fullprof.2k*	*.dat	ASCII	Only INS = 10	A

**Table 2:** Data file formats exported by *Profex* Exported data: Intensities = observed, calculated, background, difference, phase contributions. All = all intensities, *hkl* tick marks, legend, file names, axes with labels.

Format	Extension	File format	Description	Exported data
Generic ASCII	*.xy	ASCII	Generic text format with custom field separator	Intensities
*Fullprof.2k*	*.dat	ASCII	*Fullprof.2k* ‘INS = 10’ format	Intensities
Philips	*.udf	ASCII	Philips UDF format	Intensities
*Texture Plus*	*.xyp	ASCII	Input file for *Texture Plus* program (Vaudin, 2001 ▸)	Intensities
*Fityk*	*.fit	ASCII	Script for *Fityk* program version 0.9.8 (Wojdyr, 2010 ▸)	Intensities
*Gnuplot*	*.gpl	ASCII	Script for *Gnuplot* program (Williams Kelley, 2015 ▸), tested with versions 4.6 and 5.0	All
Pixel image	*.png	PNG	Pixel image of the plot display	All
Vector image	*.svg	SVG	Vector image of the plot display, to be viewed with a web browser, to be edited with a vector drawing program (*Inkscape*, *Adobe Illustrator*, *CorelDRAW* or similar)	All

## Footnotes

1 In *BGMN*’s terminology GOALs are results of the refinement or of custom calculations.
